# ACE2 Inhibits Dermal Regeneration Through Ang II in Tissue Expansion

**DOI:** 10.1111/jocd.16767

**Published:** 2025-01-20

**Authors:** Ruoxue Bai, Baoyan Liang, Yaotao Guo, Wei Liu, Zhuoyue Wen, Zhantong Wang, Yu Zhang, Jing Du, Yajuan Song, Zhou Yu, Xianjie Ma

**Affiliations:** ^1^ Department of Plastic and Reconstructive Surgery Xijing Hospital, Fourth Military Medical University Xi'an Shaanxi China; ^2^ Xijing 986 Hospital Department Fourth Military Medical University Xi'an Shaanxi China; ^3^ Key Laboratory of Aerospace Medicine of Ministry of Education School of Aerospace Medicine, Fourth Military Medical University Xi'an Shaanxi China

**Keywords:** angiotensin‐converting enzyme 2, mechanical stretch, regeneration, skin soft tissue expansion

## Abstract

**Background:**

Tissue expansion is a widely employed technique in reconstructive surgery aimed at addressing considerable skin defects. Nevertheless, matters like inadequate expansion capability and the potential for skin breakage due to the fragility of the expanded tissue present notable hurdles in enhancing skin regeneration during this process. Angiotensin‐converting enzyme 2 (ACE2) is recognized for its essential role in facilitating tissue renewal and regeneration. However, its precise impact on skin renewal during tissue expansion remains underexplored. This study seeks to elucidate ACE2's contribution to skin regeneration, specifically examining its role in collagen synthesis.

**Methods:**

This study evaluated the expression and distribution of ACE2 in expanded skin using samples derived from both rats and human patients. Additionally, we investigated ACE2 expression in stretched keratinocytes in vitro. ACE2 knockout keratinocytes were transfected with small interfering RNA (siRNA) and cocultured with fibroblasts to observe fibroblast proliferation and migration. MLN‐4760 was utilized to inhibit the ACE2 enzymatic activity. Additionally, we analyzed parameters such as the size of expanded skin, dermal thickness, and the levels of collagen I (COL I), collagen III (COL III), and transforming growth factor β (TGF‐β) to elucidate the role of ACE2 in the context of expanded skin.

**Results:**

The thinning of the expanded dermis was linked with elevated ACE2 expression. Enzymatic activity and ACE2 expression were both increased by mechanical stress. Additionally, ACE2 utilized Ang II to activate the migration and proliferation of human dermal fibroblasts. In vivo, the ACE2 inhibitor MLN‐4760 promoted skin regeneration and reduced dermal thinning by elevating COL I, COL III, and TGF‐β during expansion.

**Conclusions:**

This finding suggest that mechanical stretch increases ACE2 expression, which in turn promotes the regeneration of expanded skin. The basis for using ACE2 in clinical settings to increase tissue expansion efficacy is provided by this work.

## 1 Introduction

Skin soft tissue expansion is a widely utilized technique in reconstruction surgery [1]. This method involves placing a tissue expander benea th the normal skin and periodically injecting saline to exert mechanical stretching, thereby generating “extra” skin. The resultant surplus skin can effectively address clinical challenges such as significant defects and deformities [2]. Expanded skin offers numerous advantages, including diverse sources, consistent texture, uniform color, and optimal hair distribution while minimizing damage to the donor site [3]. Histological analyses have revealed that while the epidermis becomes thicker after expansion, the dermis may experience thinning [4]. Considering the limited regenerative capacity of the dermis, complications can arise during the process of skin soft tissue expansion. The thinning of the dermal layer can precipitate skin ulceration and potential necrosis originating from the thinner dermis. Moreover, in clinical practice, to achieve thicker and higher‐quality skin flaps, it is often necessary to prolong the duration of expander implantation, which consequently extends the surgical period for patients and exacerbates their suffering [5, 6, 7]. These issues pose significant barriers to the advancement of this technique. Consequently, further investigation into the underlying mechanisms is imperative for augmenting the performance of skin expansion and mitigate associated complications.

Expanded skin includes four sources dominated by biological growth. There are also elastic stretching, displacement of adjacent tissues, and mechanical creep. Histopathological staining of expanded skin demonstrates that the mechanical force acted on the basal cells of the epidermal layer. The epidermis was significantly thickened, and the ability of tissue regeneration was enhanced [8]. The dermis and subcutaneous tissues exhibit significant thinning, and the arrangement of collagen fibers becomes loose, yet fibroblast proliferation is increased [9, 10]. Fibroblasts are capable of synthesizing and secreting elastic fibers, collagen fibers, and various cytokines, forming collagen, elastic, and reticular fibers as well as matrix components, playing a key role in dermal tissue repair [11]. During skin expansion, despite the increased proliferation of dermal fibroblasts compared to epidermal cells, the number of cells and the amount of collagen produced still fall short of the requirements for dermal thickening. The structure of the expanded skin's dermis is disrupted, and the ratio of dermis to epidermis is imbalanced, thereby affecting the efficiency and quality of expansion. Therefore, promoting fibroblast proliferation and the synthesis and secretion of collagen is crucial for promoting dermal regeneration and improving expansion efficiency [12].

ACE2 is a transmembrane protein and a key component of the renin–angiotensin system (RAS) [13]. RAS consists of angiotensinogen, renin, angiotensin‐converting enzyme, and angiotensin II (Ang II) type 1 and type 2 receptors (AT1R, AT2R) [14]. Among them, Ang II exerts different biological effects by binding to different receptors. ACE2 can regulate the transformation and breakdown of Ang II [15]. Importantly, the RAS system is fully functional in the skin, where keratinocytes and fibroblasts synthesize renin, ACE, angiotensinogen, and receptors AT1R and AT2R [16]. Keratinocytes predominantly express AT1R, whereas fibroblasts express both AT1R and AT2R [16]. In the skin, the binding of Ang II to AT1R promotes cell proliferation and collagen production, whereas its binding to AT2R inhibits these processes [17]. Premature skin aging induced by chronic ultraviolet (UV) exposure is characterized by oxidative stress, inflammation, collagen degradation, and overall skin damage. ACE2 plays a significant role in modulating these processes. ACE2 mitigates oxidative stress by converting Ang II into Ang‐(1–7), which possesses antioxidant properties. It reduces inflammatory mediators by downregulating Ang II, a molecule that triggers inflammation through the activation of the AT1 receptor. Additionally, ACE2 inhibits the activity of matrix metalloproteinases (MMPs) through Ang‐(1–7), thereby reducing collagen degradation [18]. Additionally, ACE2 functions as a mechanosensitive protein [19]. Mechanical stretch affects cellular behavioral changes by acting on ACE2 through PKC‐βII and JNK pathways [19]. During expanded skin, mechanical stretch triggers metabolic remodeling of the skin, leading to differential changes in the epidermis and dermis, ultimately promoting skin growth. However, it remains unclear whether mechanical stretch induces alterations in ACE2 protein that affect skin soft tissue expansion, and whether changes in Ang II metabolism resulting from ACE2 modulation influence cellular function. Thus, we hypothesize that mechanical stretch induces Ang II metabolic remodeling by modulating ACE2 activity, consequently impacting skin expansion efficiency.

In this experiment, we found that ACE2 expression was upregulated in both human and rat expanded epidermis. In vitro, we demonstrate that mechanical stretch forces induce ACE2 overexpression and activation in human keratinocytes. Subsequently, through gene knockdown and coculture experiments, we established that ACE2 influences fibroblast proliferation and migration by modulating Ang II metabolism. Finally, we demonstrated in vivo that ACE2 inhibitors promote dermal collagen synthesis and mitigate dermal thinning.

## 2 Methods

### 2.1 Animal Model Establishment

Male adult Sprague–Dawley (SD) rats, weighing between 200 and 250 g, were purchased from the Animal Center of the Air Force Medical University. A total of 20 SD rats were casually categorized into two groups: the sham‐expanded group (*n* = 10) and the expansion group (*n* = 10). The rat scalp expansion model was created according to our previously described procedures [20]. To delineate the expanded region, a square area measuring 1.0 × 1.0 cm was tattooed at the center of the pre‐expanded flap. On the seventh day following tattooing, a 1‐cm diameter round silicone tissue expander (Shanghai Weining, China) was implanted under the rats' scalps. No expansion was performed in the sham‐expanded group, while the expansion group received the injection of 1‐mL sterilized saline into the expander. The first injection was administered immediately after the expander placement, with subsequent injections given every 3 days starting on day 7, until a total expansion volume of 9 mL was reached within 28 days. On day 28, 24 h following the last injection, the rats were put under anesthesia, and the enlarged skin was harvested.

### 2.2 Patient Samples

Numerous individuals provided human tissue samples, including 4 biopsies of normal skin and 11 biopsies of expanded skin. Before collection, the expanded skin samples had undergone expansion for approximately 3 months. The control group was normal skin taken from the patient. Each patient provided written informed consent to contribute the experimental sample. This study was granted by the Ethics Committee of Xijing Hospital at the Fourth Military Medical University (KY20192155‐C‐1).

### 2.3 Histological Analysis

The collected skin specimens were fixed, embedded, and sectioned. The procedure was performed as follows. The samples were placed in 4% paraformaldehyde for 24 h. Specimens were then embedded with paraffin. The samples were cut into 5‐μm thick sections and subjected to Masson's trichrome staining. The collagen volume fraction (CVF) and the thickness of the dermis were determined using ImageJ software (National Institutes of Health, Bethesda, U.S.A.).

### 2.4 RNA‐Seq

Following the methods described previously, we performed total RNA sequencing of the collected tissues [4]. Annoroad Gene Technology Co. Ltd. (http://www.annoroad.com/) performed the paired‐end RNA sequencing analysis using the Illumina HiSeq 4000 [21]. Using Bowtie2 (v2.2.5) (Maryland, USA), the sequencing reads corresponded to a reference sequence, and the RSEM software tool (v1.2.12) was used to calculate the levels of gene expression. Before analyzing downstream mRNAs, mRNAs with read counts less than 1 were excluded from the samples. The R programming language was employed to ascertain genes that exhibited differential expression.

### 2.5 Quantitative Polymerase Chain Reaction

Rat scalp samples and cells were treated to extract total RNA using TRIzol (Invitrogen, Carlsbad, CA, USA). Subsequently, the Hifair AdvanceFast cDNA Synthesis Kit (Yeasen, China) was employed to reversely transcribe mRNA into cDNA. Quantitative polymerase chain reaction (qPCR) analysis was performed using Hieff qPCR SYBR Green Master Mix (Yeasen, China) and Bio‐Rad CFX Manager 3.0. The 2^−ΔΔCt^ (threshold cycle) method was applied to quantify the target gene mRNA expression levels. The primers employed in this research are presented as follows. Rat ACE2: forward 5′‐CACCTTACGAGCCTCCTGTCAC‐3′ and reverse 5′‐GGATAACAATGCCAACCACTACCG‐3′, rat COL I: forward 5′‐TGACTGGAAGAGCGGAGAGT‐3′ and reverse 5′‐GATAGCGACATCGGCAGGAT‐3′, rat COL III: forward 5′‐TGGGCCTCAAGGTGTAAAGG‐3′ and reverse 5′‐GCCCTGGATTACCATTGTTGC‐3′, rat TGF‐β: forward 5’‐GCTGAACCAAGGAGACGGAAT‐3′ and reverse 5′‐AGGTGTTGAGCCCTTTCCAG‐3′, rat GAPDH: forward 5′‐AAGATCGGAATTAACGGATTTGGC‐3′ and reverse 5′‐GCCCTTGAAACGACCGTGAGT‐3′, human ACE2: forward 5′‐GGGATGGAGTACCGACTGGA‐3′ and reverse 5′‐GCACATCCTCCTCCCCAAAA‐3′, human GAPDH: forward 5′‐TGTTGCCATCAATGACCCCTT‐3′ and reverse 5′‐CTCCACGACGTACTCAGCG‐3′. We utilized the comparative CT method to determine the expression levels of genes, with normalization to GAPDH expression. The fold change in gene expression between two samples was calculated using the following formula: fold change = 2^(−ΔΔCT)^, where ΔΔCT = [ΔCT (gene of interest–GAPDH) for sample A–ΔCT (gene of interest–GAPDH) for sample B] [22].

### 2.6 Western Blot

To extract proteins, radioimmunoprecipitation assay buffer (Servicebio, China) was used to lyse tissue samples and cells. The bicinchoninic acid (BCA) assay kit (CWBIO Biotech, China) was utilized to measure the amount of protein. A 7.5% SDS‐PAGE gel (NCM Biotech, China) was used to separate the proteins. Then, it was transported to a PVDF membrane (Millipore, Billerica, USA). The blocking solution was prepared with tris‐buffered saline comprising 0.1% Tween‐20 (TBST) and 5% skim milk. The membranes were immersed in a blocking solution for 1 h. The membranes were then immersed at least 12 h at 4°C °C with the subsequent primary antibodies: rabbit anti‐rat ACE2 (1:1000, Abcam, United Kingdom), rabbit anti‐rat COL I (1:2000, Proteintech, China), rabbit anti‐rat COL III (1:1000, Proteintech, China), and rabbit anti‐rat TGF‐β (1:1000, Proteintech, China). After three TBST washes, the membranes were incubated for 1 h with either goat anti‐mouse (CW0102S, CWBIO, China) or goat anti‐rabbit (CW0103, CWBIO, China) secondary antibodies. Subsequently, the membranes were rinsed before being visualized by a biological imaging system (Tanon 4600, Tanon, China). Finally, relative gray scale values of the blots were calculated using ImageJ software.

### 2.7 Immunofluorescence Staining

Immunofluorescence staining was performed as previously described to detect the expression of ACE2 in expanded human and rat skins [23]. PBS containing 4% paraformaldehyde was first prepared. The tissue samples were immersed in it for about 12 h. The skin tissue was then embedded, frozen, and sectioned. After this, the following steps are performed. Sections were first blocked with PBS consisting of 5% BSA for 1 h. The primary rabbit anti‐rat ACE2 antibody (1:1000, Abcam, United Kingdom) was utilized to incubate the sections overnight at 4°C in a humidified incubator. After 12 h, the sections were incubated in the same antibody solution for 2 h at 25°C. Then, the sections were immersed in a goat anti‐rabbit Alexa Fluor 488 secondary antibody (1:200, Invitrogen, Carlsbad, USA) for 1 h at 25°C. After that, the nuclei were stained with 4′, 6‐diamidino‐2‐phenylindole (Invitrogen, Carlsbad, USA). Images were acquired using a Nikon C2 confocal microscope (Nikon, Tokyo, Japan). Subsequently, the average fluorescence intensity of immunofluorescence staining in tissue sections was measured using ImageJ software.

### 2.8 Isolation and Culture of Human Dermal Fibroblasts

Human skin was rinsed 2–3 times with PBS containing 1% penicillin/streptomycin (Gibco Life Technologies, Grand Island, USA). The skin was then cut into 2.0 × 2.0 mm pieces. The fragments were evenly distributed in the culture flasks and incubated upside down for more than 2 h. After this, 2 mL of cell culture medium was added to each flask to facilitate the migration of fibroblasts from the skin fragments. The obtained fibroblasts were subsequently transferred to culture dishes for further cultivation, with medium changes occurring every 3 days. Fibroblasts from the third passage were utilized for corresponding experiments.

### 2.9 Cell Culture

Dulbecco's modified Eagle's medium (DMEM) (Gibco, Grand Island, USA) containing 10% fetal bovine serum (FBS) (BI, Israel) was used for cell culture. Both HaCaT cells and human skin fibroblasts were nurtured using this medium. The conditions of the cell incubator were set at 37°C, 5% CO_2_, and humidified atmosphere.

### 2.10 Mechanical Stretch Devices

First, the inner wall of the stretching silicone chamber was coated with fibronectin (100 μg/mL). HaCaT cells (10^5^ cells/cm^2^) were evenly inoculated in a silica gel chamber. The stretching device (model ST‐140, STREX Corporation, Japan) was set to a 12% uniaxial sinusoidal stretching wave and 15 cycles/min [24]. In the same chambers, control cells were cultured in a static environment. After stretching, the cells and their proteins were harvested at 0, 12, 24, and 48 h.

### 2.11 Enzyme Activity Assay

ACE2 Activity Fluorometric Assay Kit (Beyotime, China) was harnessed to assess the relative enzyme activity of ACE2 in HaCaT cells cultured for 24 h post‐stretch following the manufacturer's instructions.

### 2.12 siRNA and Transfection

HaCaT cells were evenly inoculated in 6‐well plates and cultured to a 70% confluency. 20 nM negative control (NC) siRNA or ACE2 siRNA was dissolved in serum‐free medium and configured into the transfection reagents. These reagents were then introduced to the cells. The ACE2 siRNA sequences are listed below: sense strand 5′‐CCGAUCAUCUGUUGCAUAU‐3′ and anti‐sense strand 5′‐AUAUGCAACAGAUGAUCGG‐3′. After transfection for 24 h, the next procedure was performed. We extracted mRNA from the cells and examined the knockdown efficiency of ACE2 siRNA by qPCR.

### 2.13 Enzyme‐Linked Immunosorbent Assay

The Ang II enzyme‐linked immunosorbent assay (ELISA) kit (Proteintech, China) was utilized to measure the concentration of Ang II in the culture supernatants of HaCaT cells transfected with ACE2‐siRNA or NC‐siRNA following the manufacturer's instructions.

### 2.14 EdU Assay of Fibroblasts

The 5‐ethynyl‐2′‐deoxyuridine (EdU) assay was employed to estimate cell proliferation. Fibroblasts were cultured in 24‐well plates at a quantity of 1 × 10^5^ cells per well. Simultaneously, HaCaT cells were cultured in a transwell cell culture chamber (Corning, U.S.A.) with a pore size of 4 μm. HaCaT cells were pretreated for 24 h with NC‐siRNA or ACE2‐siRNA as well as 0.1% dimethyl sulfoxide (DMSO) (Sigma‐Aldrich, USA) or 10 μM of the selective ACE2 inhibitor MLN‐4760 (Millennium Pharmaceuticals, Cambridge, Massachusetts, USA). The cells were spread into the transwell chamber with the number of 3 × 10^5^ cells per well. Once the cells adhered, the transwell chambers containing HaCaT cells were placed into the wells cultured with fibroblasts for coculture over a 24‐h period, with three parallel wells established for each group. Subsequently, human skin fibroblasts were incubated with a 1 × EdU working solution (Beyotime, China) for 4 h. Cells were then fixed with PBS containing 4% paraformaldehyde for 15 min. Cells were subsequently rinsed with PBS containing 0.5% Triton X‐100 (Beyotime, China) for 10 min. Staining was performed with a 1 × Apollo staining reaction solution (Beyotime, China) in the dark for 30 min. Then, 1× Hoechst 33342 reaction solution (Beyotime, China) was added to incubate the cells for 30 min. Finally, it was blocked with an anti‐fluorescence quencher. A fluorescent microscope (Nikon ECLIPSE Ts2R, Nikon, Japan) was used to capture images of approximately six randomly selected fields from each well.

### 2.15 Cell Migration Assay

The migration capacity of cell was assessed using a transwell assay. HaCaT cells were seeded into the 24‐well plates at a density of 4 × 10^5^ cells per well and incubated for 24 h with pretreatment using NC‐siRNA and ACE2‐siRNA, along with 0.1% DMSO and 10 μM of MLN‐4760. Meanwhile, human skin fibroblasts were cultured using a transwell cell culture chamber (Corning, U.S.A.) with 8‐μm pore. Each well was evenly spread with 2 × 10^3^ human skin fibroblasts. After cell attachment, the culture medium including 2% FBS was appended to the chamber. The chambers containing human skin fibroblasts were then transferred to the plates with HaCaT cells and cocultured for 24 h, with three parallel wells established for each group. After 24 h of coculture, the fibroblasts were fixed with 4% paraformaldehyde for 15 min. Subsequently, the cells were stained with crystal violet for 20 min. Migrating fibroblasts were photographed by a microscope (Nikon ECLIPSE Ts2R, Nikon, Japan).

### 2.16 MLN‐4760 Treatment

To assess the effects of ACE2 on dermal regeneration in tissue expansion, ACE2 inhibitor MLN‐4760 was employed to treat the expanded skin. Animal models were established as previously described. The rats were segmented into two groups: the control group (CTRL group, DMSO treatment) and the MLN‐4760 group (treated with 50 μM MLN‐4760), with five rats in each group. MLN‐4760 was dissolved in DMSO solution. Both DMSO and MLN‐4760 were mixed separately with pluronic lecithin organogel and applied topically to the expanded skin at each injection [25, 26]. Rat expanded skin specimens were collected 48 h after the final expansion.

### 2.17 Statistical Analyses

At least three trials were conducted for each experiment. GraphPad Prism 9.5.1 (GraphPad, USA) was applied to assess the experimental data. All measured data were compared as mean ± standard deviation. Student's t‐test was applied for comparison between the two groups. One‐way analysis of variance (ANOVA) was employed to evaluate comparisons among multiple groups. One‐way ANOVA was followed by Tukey's test for post hoc multiple comparisons. *p* values < 0.05 (*), < 0.01 (* *), < 0.001 (* * *), or < 0.0001 (* * * *) were thought to have a statistical significance.

## 3 Results

### 3.1 Elevated ACE2 Expression in Both the Expanded Human and Rat Skin Samples Is Accomplished by the Decrease of ECM in Dermis

**FIGURE 1:**
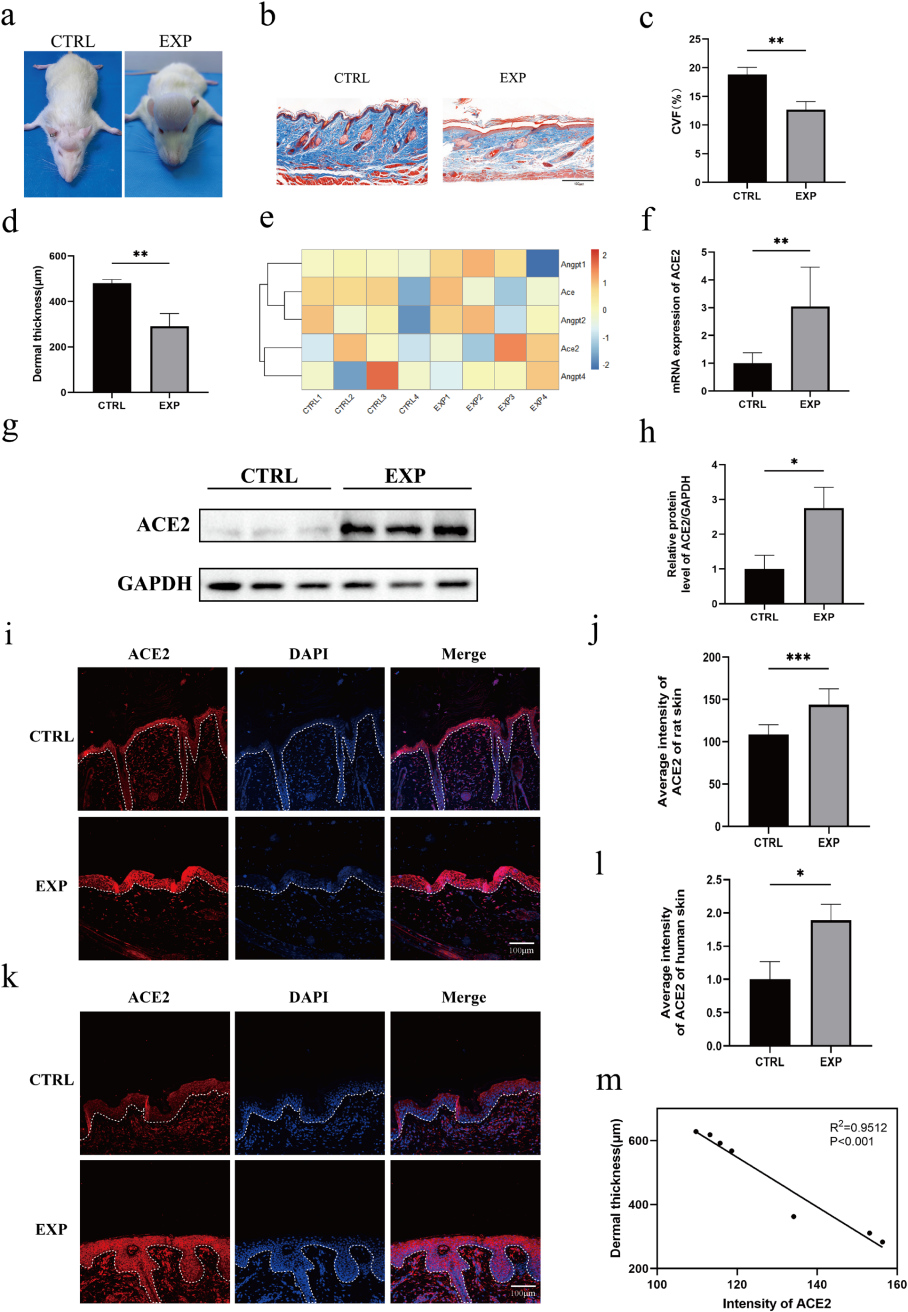
ACE2 expression in normal and expanded skins. (a) Images of rats from the control group (CTRL) and expanded group (EXP). (b) Masson's trichrome staining revealed a reduced collagen content in the expanded rat skin (×20 magnification). (c) Quantification of dermal thickness between the expanded and control skin samples (*n* = 3). (d) Quantification of the collagen content in the expanded and control skin samples (*n* = 3). (e) The heat map illustrated increased mRNA expression of ACE2 in the expanded rat skin (*n* = 4). (f) qPCR analysis confirmed the increased mRNA expression of ACE2 in the expanded rat skin (*n* = 3). (g) Western blot analysis showed the protein expression of ACE2 in the expanded rat skin. (h) Quantification of the relative protein levels of ACE2 between the expanded and control rat skins (*n* = 4). (i) Immunofluorescence labeling of ACE2 in the expanded rat epidermis. (j) Quantification of the average intensity of ACE2 in expanded and control rat epidermis (*n* = 3). (k) Immunofluorescence labeling of ACE2 in the expanded human epidermis. (l) Quantification of the average intensity of ACE2 in expanded and control human epidermis (*n* = 3). (m) Correlation analysis between the dermal ECM content and epidermal ACE2 expression. *p < 0.05, **p < 0.01, and ***p < 0.001.

Skin thickness and collagen content have been substantially changed by mechanical stretching. Figure 1a shows the macroscopic observations of rats from the expanded and control groups. The dermis of the expanded skin exhibited substantially less ECM than that of the control skin, as evidenced by Masson's trichrome staining (18.81% ± 1.24% vs. 12.70% ± 1.40%, *p* < 0.01; Figure 1b,c). Additionally, in contrast to the control group, the dermis of the expanded skin was thinner (480.04 ± 16.86 μm vs. 291.79 ± 55.48 μm, *p* < 0.01; Figure 1d).

To reveal the causal relationship between the expanded skin regeneration and the differentially expressed genes (DEGs), we analyzed the RNA‐seq data of the expanded rat skin and found the elevated expression of ACE2 in the expanded rat skin (Figure 1e). This increase in the ACE2 mRNA expression was corroborated by qPCR results (*p* < 0.01; Figure 1f). Subsequently, the elevated protein level of ACE2 in the expanded rat skin was confirmed by the Western blot analysis (*p* < 0.01; Figure 1g,h). Further, we investigated the subcellular distribution of ACE2 using immunofluorescence labeling and found that it was predominantly located in the cell membranes of keratinocytes (Figure 1i). Furthermore, compared to the control epidermis, the average intensity of ACE2 was significant higher in the expanded epidermis of rats (*p* < 0.001; Figure 1j).

Additionally, the cellular localization of ACE2 in the expanded human skin was also investigated. Immunofluorescence staining revealed that ACE2 was predominantly located in the epidermis of the expanded human skin, with minimal to no ACE2 immunofluorescence signaling observed in the dermis (Figure 1k). Correspondingly, the expanded human epidermis exhibited a significantly higher average intensity of ACE2 relative to the control skin (*p* < 0.05; Figure 1L). Furthermore, we conducted a quantitative analysis of the correlation between the immunofluorescence intensity of ACE2 and the thickness of the dermis in rat skin sections. The results demonstrated that as the immunofluorescence intensity of ACE2 increased, the dermis thickness decreased, indicating a negative correlation between ACE2 expression and the content of dermal ECM (*p* < 0.001; Figure 1m). These implied that mechanical stretch exerted by tissue expansion can stimulate ACE2 and increased ACE2 expression correlates with a decrease of ECM in the expanded dermis.

### 3.2 Mechanical Stretch Stimulated ACE2 Expression in Human Skin Keratinocyte

**FIGURE 2:**
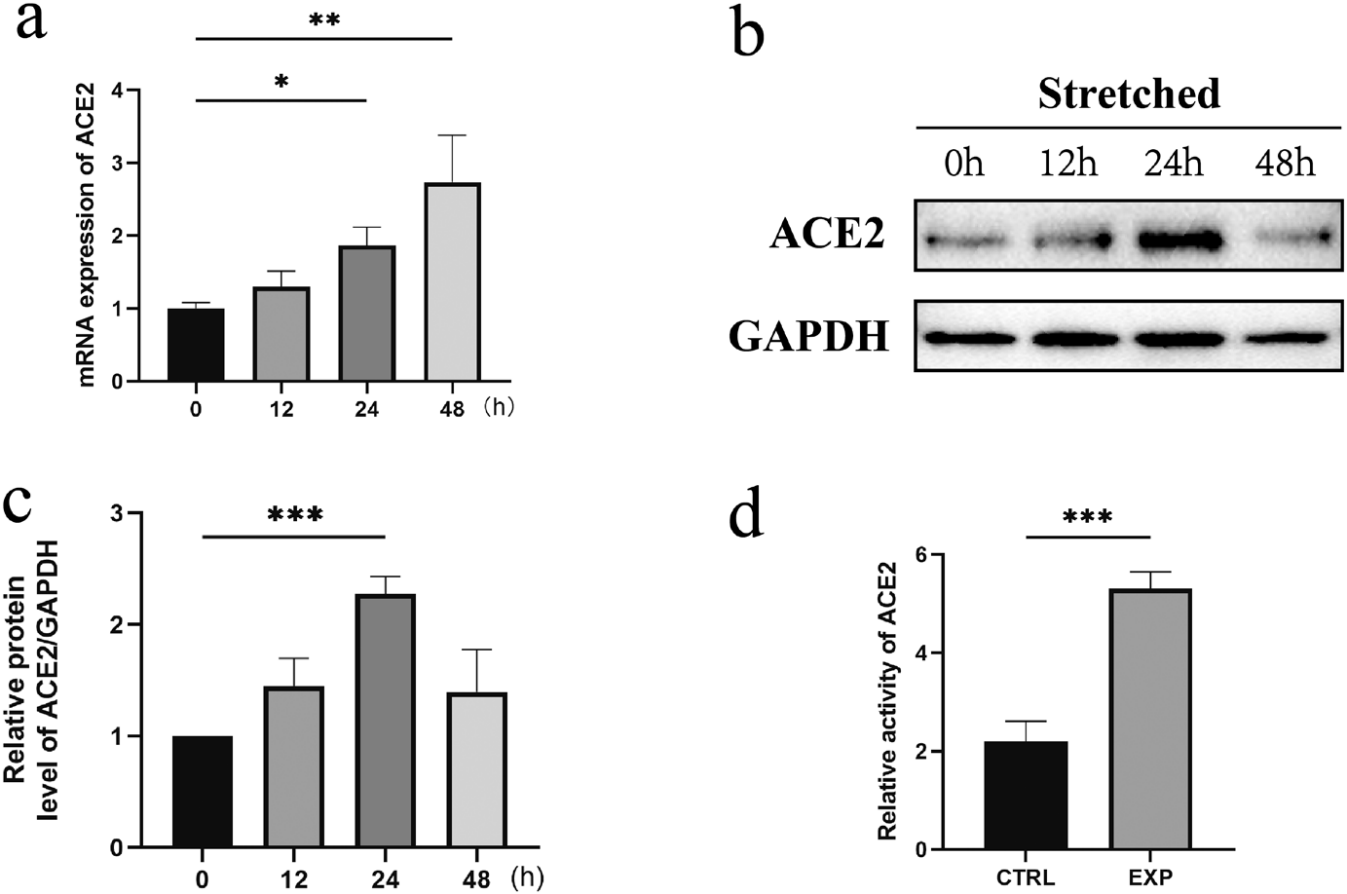
The expression of ACE2 is increased in the mechanically stretched human skin keratinocyte. (a) Mechanical stretch stimulated ACE2 mRNA in HaCaT cells. (b) Western blot analysis revealed the protein expression in stretched HaCaT cells at various time points. (c) Quantification of the relative protein levels of ACE2 at different intervals post‐stretching. (d) Comparison of the ACE2 relative enzyme activity between the expanded and control HaCaT cells. *p < 0.05, **p < 0.01, and ***p < 0.001.

To further explore the impact of mechanical stretch on ACE2 expression in vitro, mechanical stretch devices were applied to treat HaCaT cells; the results showed that mechanical stretch can stimulate ACE2 mRNA expression, and significant differences can be found at 24 h and 48 h of stretching (Figure 2a). Western blot assay demonstrated the increased ACE2 protein expression in HaCaT cells at 24 h of stretching (Figure 2b,c). Additionally, we detected the enzymatic activity of ACE2 of HaCaT cells subjected to mechanical stretch stimulation. The enzyme activity of ACE2 in the stretched cells was higher than that of the control cells (Figure 2d). Collectively, these results suggest that mechanical stretching augmented both ACE2 expression and enzyme activity of HaCaT cells.

### 3.3 ACE2 in Keratinocytes Affects the Proliferation and Migration of Human Skin Fibroblasts Through Ang II

**FIGURE 3:**
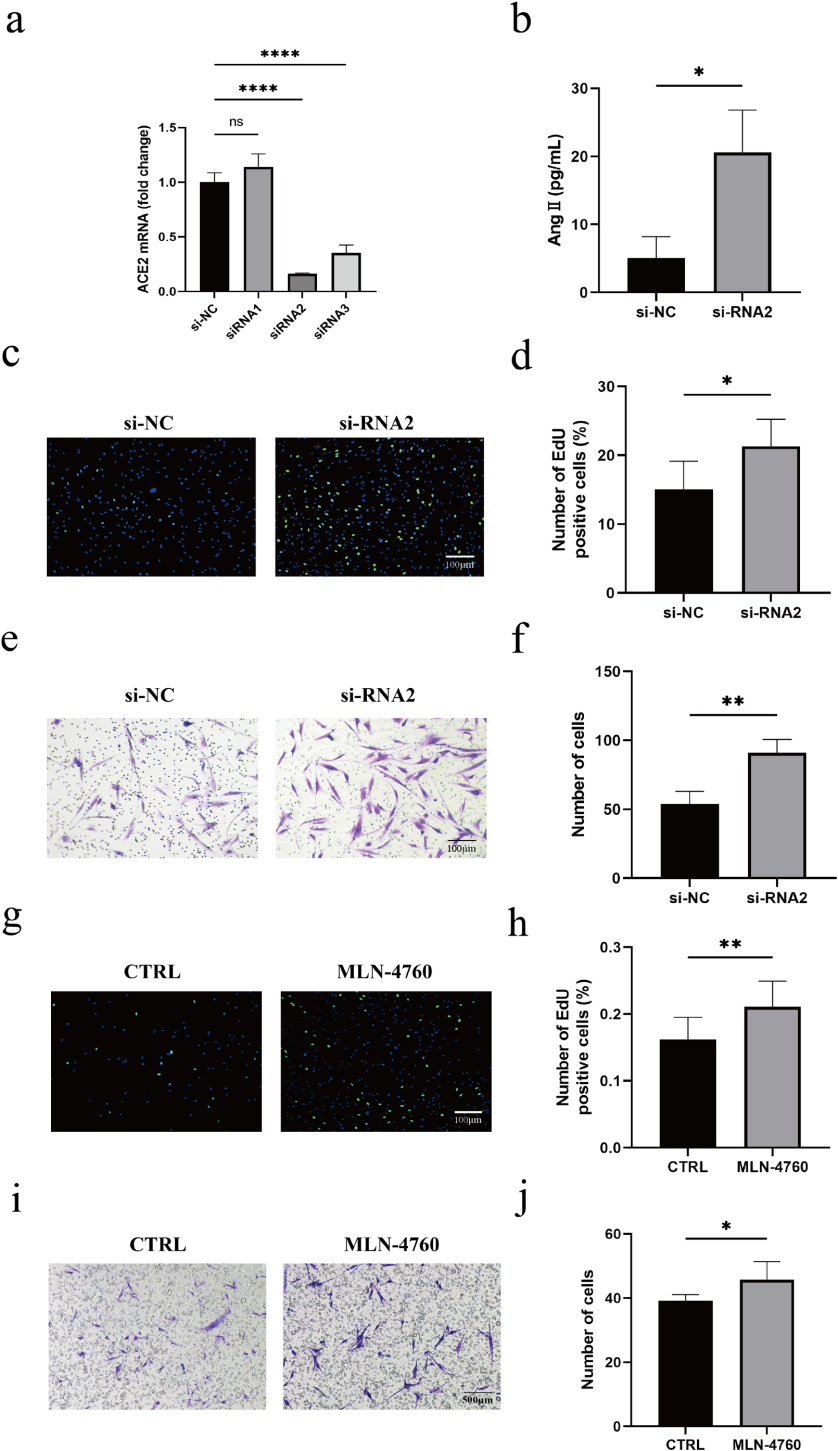
ACE2 affects the proliferation and migration of fibroblasts through Ang II. (a) ACE2 mRNA expression in negative control siRNA‐transfected HaCaT cells (si‐NC) and ACE2 siRNA‐transfected HaCaT cells (siRNA). (b) Ang II protein concentrations in the supernatants of HaCaT cells transfected with si‐NC and ACE2‐siRNA. (c) Results of the EdU proliferation assay conducted on human skin fibroblasts treated with the supernatant of HaCaT cells transfected with si‐NC and ACE2‐siRNA. (d) Proportion of EdU‐positive cells. (e) Transwell assay of human skin fibroblasts treated with the supernatant of HaCaT cells transfected with si‐NC and ACE2‐siRNA. (f) Number of migrating cells. (g) Results of the EdU proliferation assay conducted with human skin fibroblasts treated with the supernatant of HaCaT cells suffered from DMSO and MLN‐4760 treatment. (h) Proportion of EdU‐positive cells. (i) Transwell assay of human skin fibroblasts treated with the supernatant of HaCaT cells suffered from DMSO and MLN‐4760 treatment. (j) Number of migrating cells. *p < 0.05, **p < 0.01, ***p < 0.001, and ****p < 0.0001.

According to our hypothesis, the ACE2 produced by keratinocytes is integral to dermal function through its regulation of Ang II levels. To investigate the influence of ACE2 on fibroblasts, we cocultured HaCaT cells with fibroblasts. Initially, we selected the siRNA targeting ACE2 in HaCaT cells and ACE2‐siRNA2 demonstrated the highest gene silencing efficiency (Figure 3a). We then employed ELISA to assess the Ang II protein concentrations in the supernatant of HaCaT cells that were transfected with either the NC‐siRNA or ACE2‐siRNA2. The supernatant of HaCaT cells transfected with ACE2‐siRNA exhibited elevated Ang II levels compared to the NC‐siRNA (Figure 3b). This indicated that ACE2‐siRNA decreased the Ang II protein digestion of HaCaT cells. Then, we investigated the impacts of ACE2‐siRNA on fibroblasts. The results of the EdU proliferation assay illustrated that fibroblasts treated with the cell culture supernatant of ACE2 knockdown HaCaT cells exhibited significantly increased proliferation compared to the NC‐siRNA‐treated cells (Figure 3c,d). Similarly, the supernatant of ACE2‐siRNA HaCaT cells could also promote the migration of human dermal fibroblasts (Figure 3e,f). Furthermore, we explored the impacts of ACE2 on fibroblasts using the ACE2‐specific inhibitor MLN‐4760. EdU proliferation assay results showed that fibroblasts treated with the supernatant of HaCaT cells suffered from MLN‐4760 treatment, which exhibited a significantly enhanced proliferation compared to the control group (Figure 3g,h). Cell migration assay results indicated that the supernatant of MLN‐4760‐treated HaCaT cells effectively promoted the migration of human dermal fibroblasts (Figure 3i,j). These results suggest that ACE2 in the keratinocytes can regulate fibroblast proliferation and migration through Ang II.

### 3.4 ACE2 Inhibitor Benefited Expanded Rat Skin Regeneration and Rescued Dermal Thinning In Vivo

**FIGURE 4:**
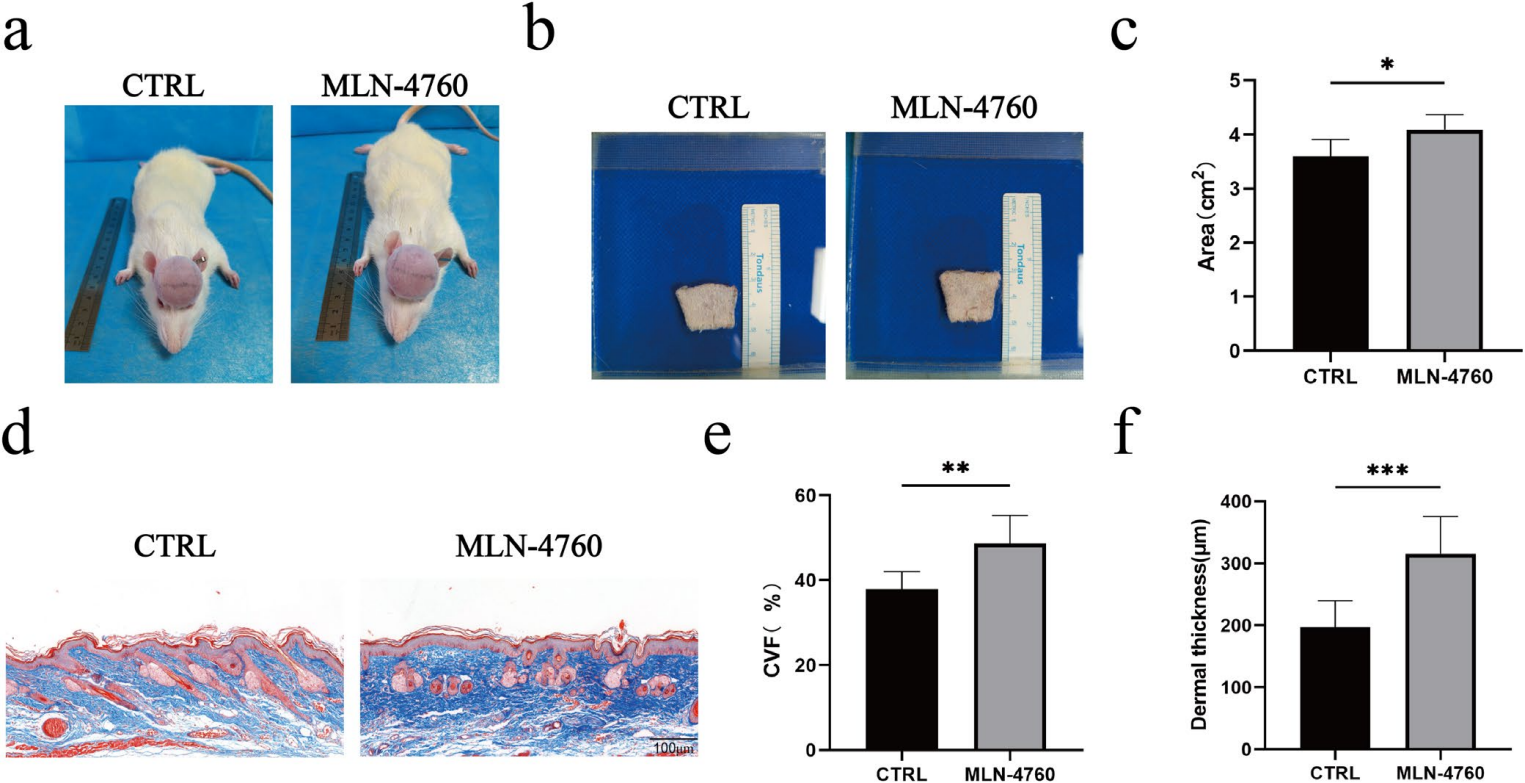
ACE2 inhibitor MLN‐4760 benefited the expanded rat skin regeneration and rescued dermal thinning in vivo. (a) Representative images of expansion areas of DMSO‐treated or MLN‐760‐treated rat skin. (b) Skin of the tattooed area after harvesting. (c) Area quantification of the expanded skin following various treatments (*n* = 3). (d) Masson's trichrome staining images of DMSO‐treated or MLN‐760‐treated skins. (e) Quantification of the collagen contents expressed as CVF following different treatments (*n* = 3). (f) Quantification of dermal thickness following different treatments (*n* = 3). *p < 0.05, **p < 0.01, and ***p < 0.001.

The rat scalp expansion model was established in accordance with established protocols. Four weeks post saline injection into the expander, the areas of the tattooed region were measured (Figure 4a,b), and the results showed that the tattooed area in the MLN‐4760 group (4.08 ± 0.27 cm^2^) was greater than that of the control group (3.59 ± 0.31 cm^2^; Figure 4c).

Furthermore, we measured both the thickness of the expanded dermis and the collagen content to investigate the influence of ACE2 inhibitor MLN‐4760 on the expanded dermis. Masson's trichrome staining indicated that the amount of ECM in the expanded skin treated with MLN‐4760 (48.62% ± 6.63%) was more than that in the control skin (37.94% ± 4.11%; Figure 4d,e). Moreover, the dermal thickness in the MLN‐4760‐treated skin samples (315.51 ± 59.93 μm) was greater compared to that of the control group (197.15 ± 42.84 μm; Figure 4f).

### 3.5 ACE2 Inhibitor MLN‐4760 Elevated the Amount of Collagens in the Expanded Rat Skin

**FIGURE 5:**
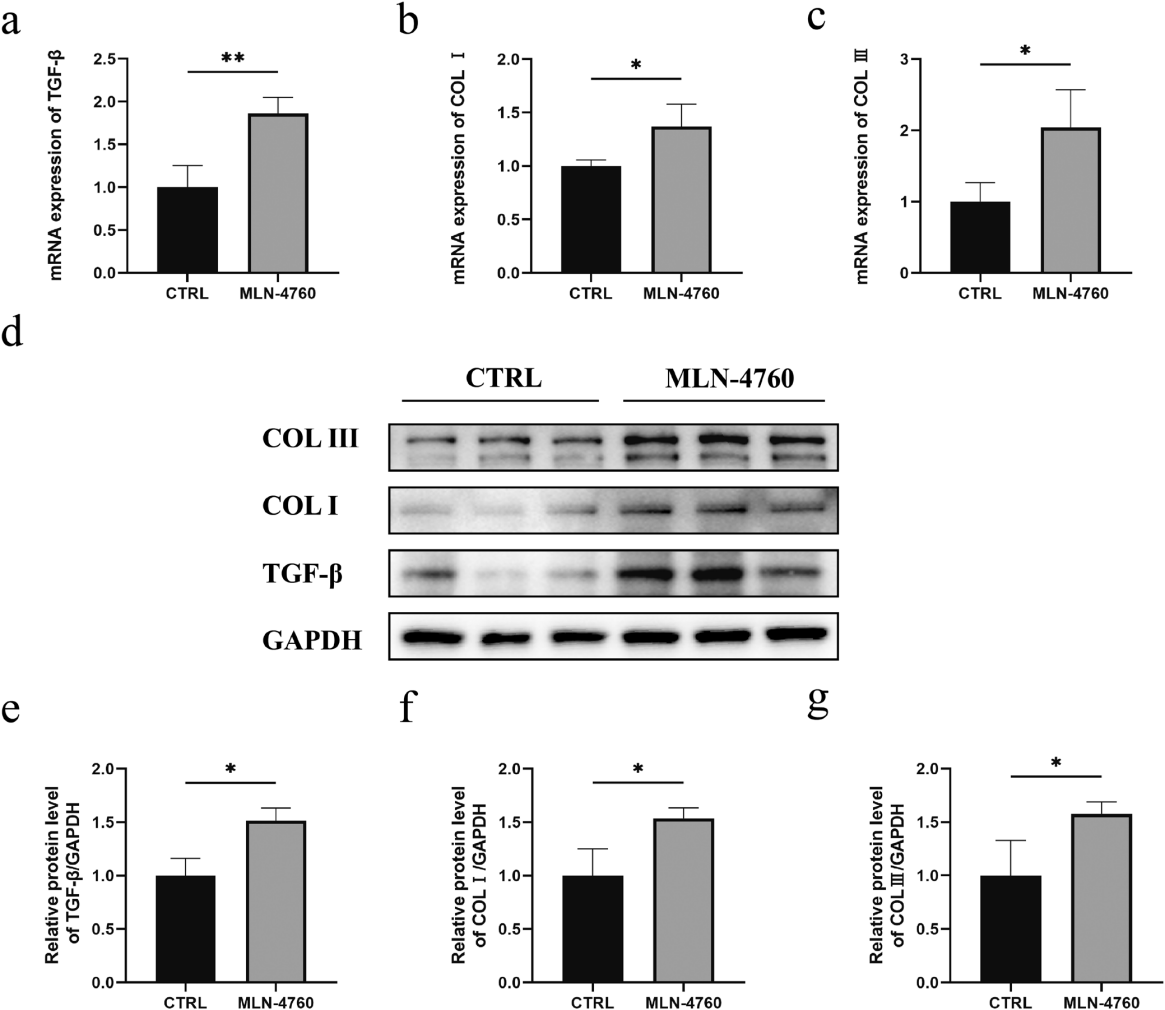
ACE2 inhibitor MLN‐4760 elevated the amount of collagens in the expanded rat skin. (a) TGF‐β mRNA expressions in DMSO‐treated and MLN‐760‐treated skins (*n* = 3). (b) COL I mRNA expressions in DMSO‐treated and MLN‐760‐treated skins (*n* = 3). (c) COL III mRNA expression in DMSO‐treated and MLN‐760‐treated skins (*n* = 3). (d) Protein levels of TGF‐β, COL I, and COL III in DMSO‐treated and MLN‐760‐treated skins detected by Western blot. (e) Quantification of the TGF‐β protein levels in DMSO‐treated and MLN‐760‐treated skins (*n* = 4). (f) Quantification of the COL I protein levels in DMSO‐treated and MLN‐760‐treated skins (*n* = 4). (g) Quantification of the COL III protein levels in DMSO‐treated and MLN‐760‐treated skins (*n* = 4). *p < 0.05, and **p < 0.01.

To examine the effect of ACE2 on the amount of ECM in the expanded rat skin, the mRNA and protein levels of COL I, COL III, and TGF‐β in the skin were tested. Compared to the control group, the mRNA levels of these genes in the MLN‐4760‐treated rat skin samples were significantly increased (Figure 5a–c). Additionally, the Western blot analysis revealed that the MLN‐4760‐treated skin exhibited elevated protein levels of COL I, COL III, and TGF‐β relative to the skin of the control group (Figure 5d–g).

## 4 Discussion

Although the skin soft tissue expansion is a fundamental technique in reconstructive surgery, the mechanisms by which it promotes skin growth remain poorly understood. This study focuses on ACE2, a key regulator of the RAS, to elucidate the molecular mechanisms underlying skin expansion engendered by mechanical stretch from a metabolic perspective. Our findings indicate that the ACE2 expression is elevated in the expanded skin, accompanied by a reduction in the amount of ECM in the expanded dermis. Additionally, we demonstrated that mechanical stretch induces ACE2 expression and activation in keratinocytes in vitro. Furthermore, inhibiting ACE2 in keratinocytes enhances fibroblast proliferation and migration by decreasing Ang II metabolism. Ultimately, the thinning of the expanded dermis was effectively rescued using ACE2 inhibitors. These findings suggest that mechanical stretch influences skin expansion efficiency through regulating ACE2‐mediated Ang II metabolism.

Mechanical stretch plays a critical role in promoting skin growth during skin soft tissue expansion [27]. Under the influence of mechanical forces, both the dermal and epidermal microenvironments undergo remodeling, leading to increased cell proliferation and skin surface area. This process was simulated using a rat scalp expansion model (Figure 1a). Notably, dermal thickness in the expansion group was markedly diminished in comparison to that in the sham‐expanded group. While dermal thinning is an essential aspect in skin soft tissue expansion, its molecular mechanisms remain unclear [28].

ACE2 is an important factor in the RAS, which can promote the breakdown and transformation of Ang II [29]. Studies have confirmed that skin tissue can synthesize the complete RAS system [30]. Under physiological conditions, RAS is associated with keratinocyte differentiation and skin sodium storage [31, 32]. Additionally, RAS plays a role in skin wound recovery, scar formation, systemic sclerosis, and dystrophic epidermolysis bullosa [32, 33]. However, the expression of RAS components varies between the dermis and epidermis. Several research have found that ACE2 is highly expressed in the basal keratinocytes of the epidermis, consistent with the findings of our study [34, 35]. We noted a significant elevation in ACE2 expression in the expanded skin of both humans and rats, suggesting its involvement in the regulation of skin soft tissue expansion (Figure 1i–l). Furthermore, ACE2 was predominantly expressed in the epidermis, with lesser expression in the dermis. The differential response of the epidermis and dermis to mechanical stimulation has posed a challenge for researchers. The high expression of ACE2 in the epidermis and the changes in ACE2 expression in the expanded skin indicate that ACE2 may be pivotal in addressing this issue.

During skin soft tissue expansion, mechanical stretch induces changes in biological processes, including voltage‐gated channel activity and cytoskeleton remodeling [4]. Mechanical stimulation serves as the driving force behind skin expansion, with mechanosensitive proteins acting as mediators that convert mechanical signals into biological responses. In previous research, we identified S100A9, CXCL1, ACTN3, ACTA1, and CKM as mechanosensitive proteins involved in the skin soft tissue expansion [4, 36]. In this study, we identified ACE2 as a mechanosensitive protein. The expression of ACE2 increased with the duration of mechanical stimulation. It has been demonstrated that mechanical stimulation regulates ACE2 expression via the JNK1/2 and PKCβII pathways [37]. Additionally, we confirmed through ELISA assay that mechanical stretch increased the enzymatic activity of ACE2.

ACE2 catalyzes the breakdown and transformation of Ang II and influences cell function by regulating the balance between Ang II binding to AT1R and AT2R. In the context of wound healing, AT1R exerts pro‐proliferative and profibrotic effects, whereas AT2R has antiproliferative and antifibrotic effects [38]. However, the specific mechanism by which ACE2 participates in the skin soft tissue expansion remains unclear. Our study illustrated that knocking down ACE2 in keratinocytes inhibited the enzymatic reaction, leading to Ang II accumulation and secretion, which in turn promotes the proliferation and migration of fibroblasts. Research has shown that Ang II activates canonical TGF‐β signaling through AT1‐R, thus facilitating cell proliferation and migration [17, 39, 40]. Our findings indicate that epidermal ACE2 protein is upregulated in the expanded skin to enhance the metabolism of Ang II and adversely affects dermal fibroblast proliferation. Furthermore, our study highlights the existence of epidermal–dermal interactions during skin soft tissue expansion. In this context, ACE2 regulates dermal growth by modulating the metabolism of the substrate Ang II. Such epidermal–dermal interactions are not uncommon in skin soft tissue expansion. Previously, we demonstrated that S100A9 protein originated from the expanded epidermis regulating dermal regeneration in a paracrine manner via promoting fibroblast proliferation [36]. This suggests a complex regulatory mechanism whereby the epidermis exerts either positive or negative influences on the dermis through distinct pathways.

In recent years, researchers have made significant efforts to enhance the efficiency of skin soft tissue expansion using methods such as bone marrow–derived stem cell transplantation, stromal vascular fraction treatment, and autologous fat grafting [41, 42, 43]. However, these approaches have notable limitations, for instance difficulty in obtaining materials and potential harm to the body. Consequently, it is essential to establish a practical and noninvasive approach aimed at improving the efficiency of skin soft tissue expansion.

Our findings indicate that the upregulation of ACE2 enzyme activity in epidermal cells is detrimental to skin growth. Consequently, we developed a novel strategy to promote skin soft tissue expansion using the ACE2 inhibitor MLN‐4760. MLN‐4760 is a potent and selective inhibitor of human ACE2 protein and has been utilized in therapeutic studies on various diseases [44, 45]. In this work, we investigated the impact of MLN‐4760 on the expansion efficiency of skin soft tissues. The results indicated that rats treated with MLN‐4760 exhibited an increased expanded skin area, enhanced dermal thickness, and elevated collagen density. Furthermore, additional experiments demonstrated that MLN‐4760 promoted the expression of COL I and COL III. These findings suggest that MLN‐4760 effectively enhances the efficiency of skin soft tissue expansion and highlights ACE2 as a promising and feasible therapeutic target to facilitate expanded skin regeneration.

In conclusion, our results demonstrate that mechanical stretch induces ACE2 upregulation in the expanded skin. And the increase of ACE2 levels in the epidermis inhibits dermal fibroblast proliferation by regulating Ang II metabolism. Furthermore, we showed that the ACE2 inhibitor MLN‐4760 effectively augments the efficiency of skin soft tissue expansion in rats. More importantly, our findings suggest a mechanistic interaction between the epidermis and dermis in the expanded skin, offering new insights into the study of skin soft tissue expansion.

## Author Contributions

R.B., B.L., Y.G., and Z.W. performed the research. X.M., Z.Y., and Y.S. supervised the research study. W.L., Z.W., Z.Y., and J.D. analyzed the data. R.B. and Y.G. wrote the paper.

## Ethics Statement

This study was granted by the Ethics Committee of Xijing Hospital at the Fourth Military Medical University (KY20192155‐C‐1).

## Conflicts of Interest

The authors declare no conflicts of interest.

## Data Availability

Research data are not shared.
